# 52528 Implementation of Web-Based Patient-Reported Outcome Measures (PROMs) in the Clinical Care of Systemic Lupus Erythematosus (SLE): A Multi-Center Prospective Cohort Study

**DOI:** 10.1017/cts.2021.770

**Published:** 2021-03-30

**Authors:** Shanthini Kasturi, Lori Lyn Price, David Curtis, W. Benjamin Nowell, Norma Terrin, Jane Salmon, Lisa Mandl, Timothy McAlindon

**Affiliations:** 1 Tufts Medical Center/Tufts University; 2 Global Healthy Living Foundation; 3 Tufts Medical Center; 4 Hospital for Special Surgery/Weill Cornell Medicine

## Abstract

ABSTRACT IMPACT: The integration of patient-reported outcome measures into clinical care is feasible and can facilitate patient-centered care for individuals with systemic lupus erythematosus. OBJECTIVES/GOALS: Patient-reported outcome measures (PROMs) are powerful tools which can facilitate patient-centered care by highlighting individuals’ experience of illness. The aim of this study was to assess the feasibility and impact of implementing web-based PROMs in the routine clinical care of outpatients with systemic lupus erythematosus (SLE). METHODS/STUDY POPULATION: Outpatients with SLE were enrolled in this longitudinal cohort study at two academic medical centers. Participants completed PROMIS computerized adaptive tests assessing multiple quality of life domains at enrollment and prior to two consecutive routinely scheduled rheumatology visits using the ArthritisPower research registry mobile or web-based application. Score reports were shared with patients and providers before the visits. Patients and rheumatologists completed post-visit surveys evaluating the utility of PROMs in the clinical encounters. Proportions with confidence intervals were calculated to evaluate survey completion rates and responses. RESULTS/ANTICIPATED RESULTS: A total of 105 SLE patients and 16 rheumatologists participated in the study. Subjects completed PROMs in 159 of 184 eligible encounters (86%, 95% CI 81 - 91), including 90% of visit 1’s (95% CI 82 - 95) and 82% of visit 2’s (95% CI 72 - 90. Patients and rheumatologists found that PROMs were useful (91% and 83% of encounters respectively) and improved communication (86% and 72%). Rheumatologists reported that PROMs impacted patient management in 51% of visits, primarily by guiding conversations (84%), but also by influencing medication changes (15%) and prompting referrals (10%). There was no statistically significant difference in visit length before (mean=19.5 min) and after (mean=20.4 min) implementation of PROMs (p=0.52). DISCUSSION/SIGNIFICANCE OF FINDINGS: The remote capture and integration of web-based PROMs into clinical care was feasible in a diverse cohort of SLE outpatients. PROMs were useful to SLE patients and rheumatologists and promoted patient-centered care by facilitating communication.

